# Analysing the Stewardship Function in Botswana’s Health System: Reflecting on the Past, Looking to the Future

**DOI:** 10.15171/ijhpm.2016.67

**Published:** 2016-06-06

**Authors:** Onalenna Seitio-Kgokgwe, Robin DC Gauld, Philip C. Hill, Pauline Barnett

**Affiliations:** ^1^Ministry of Health, Gaborone, Botswana.; ^2^Institute of Development Management, Gaborone, Botswana.; ^3^Department of Preventive and Social Medicine, University of Otago, Dunedin, New Zealand.; ^4^School of Health Sciences, University of Canterbury, Christchurch, New Zealand.

**Keywords:** Botswana, Health Policy, Health System, Stewardship, Health Legislation

## Abstract

**Background:** In many parts of the world, ongoing deficiencies in health systems compromise the delivery of health interventions. The World Health Organization (WHO) identified four functions that health systems need to perform to achieve their goals: Efforts to strengthen health systems focus on the way these functions are carried out. While a number of studies on health systems functions have been conducted, the stewardship function has received limited attention. In this article, we evaluate the extent to which the Botswana Ministry of Health (MoH) undertook its stewardship role.

**Methods:** We used the WHO Health Systems Performance Assessment Frame (HSPAF) to guide analysis of the stewardship function of the Botswana’s MoH focusing on formulation of national health policies, exerting influence through health regulation, and coalition building. Data were abstracted from published and unpublished documents. We interviewed 54 key informants comprising staff of the MoH (N = 40) and stakeholder organizations (N = 14). Data from documents was analyzed through content analysis. Interviews were transcribed and analyzed through thematic analysis.

**Results:** A lack of capacity for health policy development was identified. Significant policy gaps existed in some areas. Challenges were reported in policy implementation. While the MoH made efforts in developing various statutes that regulated different aspects of the health system, some gaps existed in the regulatory framework. Poor enforcement of legislation was a challenge. Although the MoH had a high number of stakeholders, the mechanisms for stakeholder engagement in the planning processes were weak.

**Conclusion:** Problems in the exercise of the stewardship function posed challenges in ensuring accountability and limited the health system’s ability to benefit from its stakeholders. Ongoing efforts to establish a District Health System under control of the MoH, attempts to improve service delivery at a national level and political will to strengthen public-private engagement mechanisms are some of the prospects that can improve the MoH’s stewardship function.

## Introduction

In many parts of the world, particularly in developing countries, ongoing deficiencies in health systems compromise the delivery of health interventions necessary for attainment of good health outcomes for their populations.^1^ In 2000, the World Health Organization (WHO) identified four functions that health systems need to perform to achieve their goals: service delivery, health financing, resource generation, and stewardship.^1^ These functions are viewed as levers of health systems performance. Efforts to strengthen health systems, therefore, focus to a large extent on the way these functions are carried out. Through the Ouagadougou Declaration, the WHO Africa Region, identified the need to focus on health system functions among its key priorities that will foster implementation of primary healthcare (PHC).^2^ The WHO European Region also articulated the importance of health system functions in its Tallinn Charter.^3^ While a number of studies on health systems functions have been conducted, the *stewardship* function has received limited attention to date, and is poorly understood^4,5^ despite its centrality to health system performance.

*Stewardship* is a term that is often used interchangeably with *governance*^5,6^ which is a much more widely used and understood concept probably because of the work of international agencies such as the World Bank,^7^ the United Nations Development Program (UNDP),^8^ and the Institute on Governance^9^ that ensure governance remains topical in their policy agenda. At a broader level, the definition of governance focuses on political and public administration of societies. This includes how important policies and decisions are made, the role of the populace in decision-making, and the institutions and mechanisms through which governments are held accountable.^7-9^

The concept of governance or stewardship in the health sector is underdeveloped and literature in this area is limited.^5,10^ Saltman and Ferroussier-Davis define stewardship as:

*“…a function of government responsible for the welfare of the population, and concerned about the trust and legitimacy with which its activities are viewed by the general public.”* ^11^

The United States Agency for International Development (USAID) posits that the objective of health system governance is to protect and promote health.^12^ Key governance activities are cited as setting strategic direction and objectives, making policies, laws, rules, regulations or decisions, raising and allocating resources to accomplish objectives and ensuring that the strategic objectives are accomplished.^12^

The WHO views stewardship, also referred to as leadership and governance, as the most important health system function because of the influence it has on other functions.^1^ While the contribution of various actors in managing the well-being of populations is acknowledged, ministries of health can assume more stewardship responsibilities on behalf of their governments.^1^ Undertaking this huge responsibility with limited evidence to guide the action of health ministries is a daunting task. Drawing from a larger research study which assessed the performance of the Botswana Ministry of Health (MoH) using the WHO Health System Performance Assessment Framework (HSPAF),^13^ this paper analyses the stewardship function of Botswana’s health system. Specifically, it seeks to: (*i*) evaluate the performance of the stewardship function by the MoH; and (*ii*) identify opportunities for enhancing the ability of the MoH to undertake its leadership role with respect to stewardship.

## Methods

### Conceptual Framework

Building on the work of the WHO, the World Bank and other international agencies working in the area of governance, a number of authors and organizations have proposed and used different frameworks to study stewardship in health systems. Table 1 presents a summary of some governance frameworks from the literature.

**Table 1:** Dimensions of Health System Governance

**Author**	**Dimension of Governance**
USAID	Overall governance (WGI): • Voice and accountability • Political stability • Governance effectiveness • Rule of law • Regulatory quality and control of corruption	Health specific indicators: • Information and assessment capacity • Policy formulation and planning • Social participation and system responsiveness • Accountability and regulation
Siddiqi et al	• Strategic vision • Participation and consensus orientation • Rule of law • Transparency • Responsiveness	• Equity and inclusiveness • Accountability • Intelligence and information • Ethics-respect for autonomy
Mikkelsen-Lopez et al	• Strategic vision and policy design • Participation and consensus orientation • Accountability	• Transparency • Corruption

Abbreviations: WGI, World Governance Indicators; USAID, the United States Agency for International Development.

This paper uses the WHO framework^1,14^ for two reasons. First, it is gaining significant popularity in health system strengthening studies and activities in developing countries because of its comprehensiveness in assessing overall and specific aspects of health system performance across countries with diverse backgrounds. Second, the WHO framework is produced for and on behalf of its member countries, which is effectively almost every country in the world. The WHO identified three main tasks of stewardship as: (*i*) formulation of health policy to define the vision and set strategic direction; (*ii*) exerting influence through regulations, and (*iii*) collecting and using intelligence.^1^ Travis et al expanded these tasks to include building and sustaining partnership, creating a fit between policy objectives and organizational structure and culture, and ensuring accountability.^15^ Building on this work and improving on its earlier view of stewardship, the WHO states that:

*“Leadership and governance involves ensuring strategic policy frameworks exist and are combined with effective oversight, coalition-building, the provision of appropriate regulations and incentives, attention to system-design, and accountability.”* ^14^

Based on this perspective, we assessed the stewardship function of the Botswana’s MoH focusing on: (*i*) formulation of national health policies, (*ii*) exerting influence through health regulation, and (*iii*) coalition building. Under each of these subfunctions, specific assessment elements were identified and used to guide an analysis (Table 1).

The guiding principle in identifying the assessment elements was relevance and ability to provide actionable feedback to policy-makers. The system-design and collecting and using evidence are addressed in different publications.^16,17^

National health policies define the vision and set strategic direction for the health system, in so doing they establish consensus among the different actors.^1,14^ Effective leadership is critical for policy development and provision of oversight in the health system.

Health systems comprise many players with different interests. The role of government is to influence the behaviours of these actors to protect the well-being of the population through regulation.^15,18^ Effective regulation depends on the extent to which enforcement is monitored.^18^

Building partnerships is considered as a way of exerting influence in a less formal way than regulations.^15^ Establishing and managing effective partnerships requires setting up structures and mechanisms to facilitate partner engagement.^19,20^ Effective leadership is essential for guiding and supporting the activities of partnerships to optimize their contribution for health system development.

### The Setting

Botswana has an estimated population of 2 million.^21^ The country’s health system is financed through a mix of sources. The government accounts for about 67% of the total health expenditure while private sources and donors account for 21% and 12%, respectively.^22^

The public health system is organised into different levels with mobile stops and clinics representing the lowest level, while the three referral hospitals are the highest level.^23,24^ Before April 2010, responsibility for public health services was shared by the MoH and the Ministry of Local Government (MLG). The role of the MoH was to provide policy direction and leadership. The MoH was also directly responsible for all public hospitals. PHC services were decentralized to the MLG and managed by health departments in various districts and town councils since the 1970s.^25^

The private health sector comprises both not-for-profit and for-profit health facilities including pharmacies and medical laboratories. There are three main Medical Aid Schemes that play a significant role in financing private healthcare.^26,27^ Other key players in the health system in Botswana include international agencies such as WHO, The United Nations Children’s Fund (UNICEF), USAID, and United Nations Development Programme (UNDP); donor agencies; and other countries through bilateral and multilateral partnerships.

### Study Design

The larger study adopted a case study approach^28^ and used a mixed methods research design to assess performance of the Botswana MoH.^13^ The dominant methodology was qualitative using document analysis, key informants and focus group interviews. The quantitative arm comprised surveys for hospital managers and health workers.^13^ Both qualitative and quantitative strands of the study occurred simultaneously, answering related aspects of the study questions.^29,30^ This article is based on qualitative data from document analysis and key informant interviews.

### Data Collection

Data were collected by the first author between November 2009 and December 2011.

#### Document Analysis

Data was extracted from published and unpublished documents which included National Development Plans, MoH strategic and annual performance plans and reports, Government and MoH policies, and related reports from other agencies such as the WHO using the indicators in Table 2 as a guide. We searched electronic databases such as PubMed, Science Direct, Ovid, Scopus, Web of Knowledge, ProQuest, and Google Scholar for published articles on different aspects of stewardship in Botswana health systems. The key words and phrases included Botswana Ministry of Health AND stewardship; leadership AND Ministry of Health, Botswana; Governance AND Botswana Ministry of Health; health policy AND Botswana; health regulation AND Botswana; partnerships AND Ministry of Health Botswana. Related articles were also tracked from the main searches.

**Table 2:** Assessment Areas and Elements for Evaluating the Stewardship Function of the Botswana MoH

**Assessment Areas**	**Elements**
National health policies	Availability of leadership capacity for policy development Availability of national health policies Adequacy of policy implementation
Health regulation	Availability and adequacy of health legislation Adequacy of enforcement and monitoring adherence to available legislation
Coalition building	Availability of mechanisms for engaging stakeholders in planning and delivery of health services Extent of stakeholder engagement in health policy and planning

Abbreviation: MoH, Ministry of Health.

#### Key Informant Interviews

Fifty-four participants were purposefully selected and interviewed on the basis of the relevance of their positions to the study questions.^13^ These included policy-makers, senior management and staff of the MoH (n = 40), including a total of 9 retired employees identified through a snowballing technique.^31^ A total of fourteen (n = 14) senior officers from various stakeholder organizations such as the MLG, mission hospitals, regulatory bodies, professional organizations and international agencies working in Botswana were also part of the sample. Participants were recruited through personal contact. An information package comprising request for participation, information sheet detailing the objectives of the study and the participants’ rights, and the consent form was issued to participants.^13^ An interview guide using assessment areas and indicators identified in Table 2 was used in data collection. Interviews were conducted until data saturation was reached. All participants approached agreed to participate in the study. Interviews were conducted in English, which is the official language in Botswana; tape recorded and transcribed by the first author with the assistance of some research assistants.

### Data Analysis

Data from documents and interview transcripts were analysed using content and thematic analysis, respectively, guided by Miles and Huberman’s approach which consists of data reduction, data display and conclusion drawing/verification.^32^ A deductive approach was adopted where the study indicators acted as the organizing framework. The key informants were categorized into two main groups coded as ‘M’ for MoH employees and ‘S’ for participants from stakeholder organizations. The MoH category was further divided to designate retired officers and these were coded as ‘MR.’

## Results

### National Health Policies

#### Leadership Capacity for National Health Policy Development

A lack of capacity for health policy development was identified through both self-assessment of the MoH^33^ and a review by external consultants, which noted that the approach was incoherent, fragmented and weak.^34^ The leadership of the MoH through policy guidance was observed to be limited:

*“The MoH, represented by its Headquarters, has the prime responsibility for the development of policy (congruent with central government responsibility) and for providing strategic direction. However, the central steering on policy development is not always visible.”* ^35^

These observations were supported by key informants who cited the lack of leadership skills as a major impediment to provision of strategic direction to the health sector:

*“From a leadership perspective, based on the competencies and strengths, and the skill sets that the Ministry has at this time, one would argue that they actually do not have the full repertoire of skills for leadership…because of that there has been a lack of a vision. We are still functioning on a reactionary basis…”* (S13).

These participants also noted that due to limited leadership capacity at the MoH headquarters, there is overreliance on external assistance creating a situation where the health system is controlled from outside:

*“If you are trying to lead a national health system, a part of that leadership is who you use to lead. If you are led mainly by people coming in as consultants from outside, then you are not leading…you are being led from outside. So the thinking is not generated from within the system. Leadership is about, do you have the people who can lead? Is the Ministry leading or it is just bringing people saying ‘oh help me do this, help me do that’…”* (S1).

#### Availability of Health Policies

The national health policy that was in use in 2009-2011 was developed in 1995. While this policy defined the responsibilities of the MoH emphasizing its leadership role in particular, and that of the MLG in terms of providing PHC,^36^ its implementation was limited. Several participants cited its lack of explicitness and absence of a plan to facilitate its implementation as some of the major challenges that limited its use:

*“Ideally where we have a National Health Policy, in my opinion we should have an implementation master plan that can be used to be able to monitor the implementation of this policy and also to see if we are meeting our set targets or milestones…”* (M13).

With many changes affecting the health system, this policy was deemed limited and out-dated and consequently was under review. Significant policy gaps were identified in some areas that are corporate in nature such as quality and performance of public and private health facilities, and health financing, including the role of Medical Aid Schemes. Rapid turnover of senior management and political leadership, and inadequate expertise in policy development were cited as some of the factors that contributed to lack of policies or delays in the policy development process:

*“We spend a lot of time developing them (policies). When it comes to implementation, there is a lot of delay…the high attrition rate does not help the situation because the new senior managers who come in…be it the Permanent Secretary or the Minister…they need time to study the policy and pass it, and sign it for implementation. By the time they are just about to sign it for implementation, they are on their way out…”* (MR40).

While there were challenges in corporate level policies, the MoH through the support of partners had done relatively well in developing policies and guidelines for different technical aspects. Examples of areas that were well-addressed in terms of policies included sexual and reproductive health, child health and nutrition, HIV/AIDS, and other communicable diseases such as malaria and tuberculosis (TB).

#### Adequacy of Policy Implementation

Considerable challenges were reported in the area of policy implementation. Of particular concern was the ability of the MoH to ensure policy compliance in the delivery of health services by the MLG and the private health sector. For delivery of PHC services, responsibilities and relationships between the two ministries were poorly defined and inadequately guided by policy. Consequently, misunderstanding and conflicts were reported.^37^ In the absence of policies to guide relationships between the two ministries, several participants indicated that the MoH used measures such as training and support visits to promote implementation of policies and adherence to required standards. Good interpersonal relationships were cited as critical in enabling the MoH access to PHC services:

*“We had to make sure that we establish relationships…Every time there was a change of officers one would have to start all over again establishing interpersonal relationships until trust is built. Otherwise things will just go haywire…”* (MR37).

The structures essential for ensuring implementation of national health policies by the MLG were weak. Although a PHC Support Unit in the MoH Department of PHC, and a committee established to coordinate the MoH and the MLG (PHC Coordinating Committee) were responsible for facilitating policy implementation in the districts, these structures were constrained in many ways:

*“…the mechanisms in place at the central level have not worked effectively either. Terms of Reference for the PHC Coordinating Committee were not drawn at all…The Status of the committee has not been supported by any statutes, leaving room for participatory discretion on both parties as well as on the committee membership and its level and frequency of participation.”* ^34^

The PHC Support Unit lacked the capacity to carry out supports visits as planned^37^ while the PHC Coordinating Committee was rarely functional:

*“…it (the Committee) was there but not meeting as regularly as it should have been. It was not providing the leadership it should have been providing. It was not looking at issues that have relevance to policy…Obviously if the structure doesn’t meet, nobody is bringing the challenges and barriers that are there operationally, some being system issues, some being issues of personalities I have to say…and things continued to deteriorate…”* (MR34).

The private sector was another area in which the stewardship role of the MoH was challenged. While the MoH had developed a number of technical policies, these did not filter through to the private health sector. Examples of disease treatment policies issued by the Ministry which failed to influence the practice of private health providers were cited and the capacity of the Ministry to provide oversight was questioned:

*“I would like to see a situation where all conditions in the country are managed the same way, for example, control of diarrheal diseases. It should not matter where a child goes. Whether the child goes to the private sector or the public sector, it should not make a difference. The care should be the same. But this, we were never able to impose so to speak…”* (MR32).

*“It almost seems that the Ministry of Health is too weak to enforce, or it has not structured within itself enforcing entities to ensure that the private sector works according to what ought to be done. Look at the issue of what medications should be used-we’ve got upsurge on MDR TB (multi-drug resistant tuberculosis), the reason is that we are using second line TB treatment in the private sector. Once people get TB they already have resistance…”* (S1).

The only exception was in the area of HIV/AIDS where national treatment protocols were used in both the public and private sectors. The motivating factor was the move towards public-private partnerships where private practitioners were providing HIV/AIDS services for public sector patients at a cost to the Government.

### Health Regulation

#### Availability of Health Legislation

The MoH made considerable efforts in facilitating the development of various statutes that regulated different aspects of the health system (Table 3).

**Table 3:** Selected Health Legislation in Botswana (2010)

**Act/Regulation**	**Date**	**Purpose**
Public Health: Chapter 63:01	1981	Provide for notification and control of certain diseases subject to the international health regulations; regulate sanitation and housing; provide for the protection of foodstuffs and water supplies; and generally to make provision for public health.
Private Hospitals and Nursing Homes Act	1989	Regulate private hospitals, nursing homes, and similar institutions.
Drugs and Related Substances Act No. 18 of 1992	1992	Provide for the control and regulation of all drugs imported into, or exported from Botswana.
Statutory Instrument No. 46 of 1993: Drugs and Related Substances Regulations, 1993	1993	Establish the Drug Advisory Board; defines procedures and processes related to registration, distribution, prescribing and dispensing of drugs.
Botswana Health Professions: Chapter 61:02	2001	Regulate and control the practice and training of medicine, dentistry, pharmacy, and allied health professions.
Nurses and Midwives: Chapter 61:03	1997	Provide for the regulation of the practice of and training of nursing and midwifery in Botswana.

Source: Government of Botswana various statutes.

In view of the epidemiological and other changes affecting the health system, the Public Health Act^38^ was observed to be limited and outdated by most participants and, consequently, it was under review.

The Private Hospitals and Nursing Homes Act^39^ jurisdiction did not include private clinics. It was also limited in addressing standards and quality issues in private establishments:

*“The Private Hospitals and Nursing Homes Act is very, very weak…It is very minimal on what is required. What we need are detailed regulations detailing what is expected…”* (M17).

The Drugs and Related Substances Act^40^ covered both the public and private sector. Botswana Health Professions Act^41^ regulated the practice of all health professionals practising in the country except nurses and midwives who were regulated by the Nurses and Midwives Act.^42^

While participants acknowledged the Ministry’s efforts in ensuring availability of health legislation, they also noted some gaps in the regulatory framework. These included obsolete statutes and lack of legislation to guide some key policy areas such as handling of human tissue which became important with the establishment of the Medical School; establishing health facilities; maintaining quality assurance in public facilities; and general issues of hospital performance:

*“There is no legislation which deals with government hospitals…At the moment there is no requirement for government hospitals to be licensed or even to be accredited…Government can build hospitals of any standard where they like…”* (M17).

#### Adequacy of Enforcement and Monitoring Adherence to Available Legislation

While most participants acknowledged the MoH’s efforts in developing laws and regulations, almost all participants cited poor enforcement as a major challenge. The regulatory enforcement entities such as the Drug Regulatory Unit and the National Drug Quality Control Laboratory were limited in capacity and hence unable to fulfil their mandate.^43,44^ With respect to the Drug and Related Substances Act, participants noted:

*“That is a very well-meaning law…the gap now becomes the resources that we have within the Drug Regulatory Unit…It has been very difficult for that function to be adequate for the whole country…There is nobody who actually goes around doctors’ surgeries and pharmacies to make sure that the drugs that are used are actually safe…”* (MR40).

“*…as it is now, there is a very big gap. The drugs that are actually being used in our market, nobody actually goes out there to sample if they are of good quality or whether there are any issues there…But drugs are such a crucial commodity, somebody ought to be doing that. But there is no capacity*…” (S11).

The health professions’ regulatory bodies also faced similar challenges of capacity, creating regulatory problems. Lack of knowledge of legislation affecting healthcare delivery among managers was another factor limiting enforcement and adherence to legislative requirements:

“*Nobody is aware of the regulations or laws. We are just working. Even people who are responsible for enforcing them, they don’t know. They get to know when there is something they have to attend*…” (M28).

“*We may be having the instruments. We are not enforcing them every day. We are not educating the communities or the people about these instruments every day. It is only when there is a problem, we go and read quickly and say ‘I can use this, or I have a gap, I need to develop an instrument to address this issue’…”* (MR35).

### Coalition Building

#### Mechanism for Engaging Stakeholders in Planning and Delivery of Health Services

The MoH was observed to have a high number of stakeholders working in different areas in the health sector. It was, however, noted that the mechanisms for engaging these stakeholders were weak. The lack of a forum that could bring stakeholders together to promote a coordinated approach to stakeholder contribution to health sector development was cited as a key weakness:

“*Each organization would meet the Ministry management to probably tackle that particular section of their core business. But really bringing all of them together so that we probably think together on how and what is the future of our health services, that has been quite a big challenge…”* (MR40).

Some participants noted past unsuccessful efforts to establish a stakeholder forum. For the private sector, participants cited the existence of the High Level Consultative Committee (HLCC) which brought in the private sector players including practitioners and suppliers of various health commodities. The HLCC met quarterly and was chaired by the Minister of Health. Although it could act as a good platform for harnessing private health sector contribution, HLCC was viewed to have limited focus on issues of strategic importance. The private sector was also expected to be represented by Botswana Confederation of Commerce, Industry and Manpower (BOCCIM-now called Business Botswana), a national body that stood for the interests of its members in negotiating business opportunities with government. Performance of BOCCIM was also perceived to be limited:

*“…the private sector is supposed to coordinate itself through BOCCIM. BOCCIM is supposed to be the voice of the private sector going into government, engaging with government. But at the moment, the structure within BOCCIM is not where it should be…some people still want to report individually to the Ministry…” (S11).*

#### Extent of Stakeholder Involvement in Health Policy and Planning

In general, participants cited inadequate involvement of stakeholders in planning for the health sector particularly at a strategic level. They felt that stakeholders are usually asked to contribute to plans very late in the planning process:

*“What I have realised over the years is that the Ministry prefers to start internally…but only using resources that it has got internally and then here and there, they then call a stakeholder input workshop after the document has been put together or probably at the time of implementation, which…I think is quite late to actually include your stakeholder…by the time you come in, it is very difficult for your input to influence the strategy…”* (MR 40).

Despite the weaknesses in engaging stakeholders in the planning process at the strategic level, several participants acknowledged the contribution of development partners and other international agencies that provided technical assistance at program level. Notwithstanding this support, some participants noted the challenges associated with technical assistance in the absence of local capacity. The lack of appropriate skills and competencies at the technical level where choices about programs were made was considered a major limitation in ensuring that policies and plans were nationally driven and address national priorities. The ability to effectively engage in dialogue with technical experts was viewed as essential:

*“…we have this huge divide between those coming with technical expertise and those on the ground. If the gap is too wide…it simply means that the agenda becomes fully driven by those coming from outside because there is nobody local who is able to balance and ‘say we want you to go in this direction,’ so you end up taking things wholesale…”* (S1).

*"…you do need the human resource capacity and skills to help you make appropriate and relevant choices…you need to be able to make sure you have those skills in place so that when you are getting external expertise and advice, you are interacting at almost the same level. If there is a very big difference in terms of the level of expertise between the international expertise you need to bring to guide certain processes, and the ones having to make decisions on how to move forward in terms of strategy, you can then be influenced by outside thinking…”*(MR33).

While generally partners from the United Nations (UN) family such as the WHO, UNICEF, UNDP, and others were found to fully support national priorities, participants acknowledged that other partners may have different and unrelated agendas which require effective management to strike an acceptable balance:

“*When you get many other kinds of partners also, the partners of course have their own desires and needs in any partnership…you need to have the right kind of staff capacities in place to make sure that within the context of that partnership, your national needs are the ones that are met first*…” (MR33).

Generally, participants felt that the MoH was not fully exploiting the capabilities of its stakeholders to strengthen the health system. Inadequate management of the stakeholders was an ongoing concern. Several informants emphasized the need for strong leadership and commitment at the highest level of the Ministry in the area of stakeholder management to optimize stakeholder contributions and support for national health objectives:

*“… you need the accounting officer to also find passion in that because it means calling together the heads of different missions and making sure that you have a proper and common understanding…the reason why it needs to be driven by the accounting officer is the fact that it also includes saying ‘no’ to assistance that is not consistent with your plan…” (MR34).*

## Discussion

In this article, we assessed the performance of the stewardship function by Botswana MoH. We used the WHO’s concept of stewardship to provide a framework for this analysis focusing on formulation and implementation of national health policies, exerting influence through health regulation, and coalition building. This section summarises the findings of the analysis and explores opportunities for MoH to improve its stewardship function.

### National Health Policy Development and Implementation

Chronic stewardship challenges were observed in Botswana’s health system. This was related mainly to limited human resource capacity at leadership and technical levels, weak oversight structures and lack of appropriate policy tools to influence and guide policy implementation. Consequently, oversight of key health system activities such as delivery and quality of health services provided by other stakeholders including MLG and the private sector was inadequate.

The oversight challenges in provision of PHC services provided by MLG were largely related to the nature of decentralization. The MoH devolved responsibility for delivery of PHC to the MLG within a very weak policy and regulatory framework.^37^ While central level control over allocation of resources has been used in countries with decentralized health systems such as Zambia,^45^ the MoH in Botswana had no influence on resource flows to the districts which received their funding from the central government through the MLG.

Challenges in stewardship have been reported in many health systems with fiscal decentralization.^45,46^ Reports of local preferences which differed from national priorities, and the limited capacity for the central level to influence such divergent behaviours have been cited in these health systems. Strong central level planning approaches that define priorities and set targets, formal contractual agreements that ensure accountability in implementation of national policies,^47,48^ and effective monitoring and reporting^49^ are other mechanisms used by ministries of health to encourage local authorities to deliver on national priorities. These approaches are, however, still limited in Botswana, as reported in this article and elsewhere.^13^

The MoH also faced significant challenges in influencing the behaviour of the private providers who have grown numerically and in diversity within a very limited policy framework that has failed to adequately influence the quality of services they provided to ensure that these services addressed national priorities. The lack of oversight in this sector is a major concern considering the view that the private sector plays a greater role than was previously thought in many developing countries where it serves all population groups, and not only the affluent.^50-52^ Market failures inherent in private provision of health services require more intervention on the part of the MoH to ensure public safety and adherence to national policies.

While in other countries policy options such as use of financial incentives in the form of funding and subsidies, negotiations, and effective monitoring and reporting^52,53^ are used to promote private sector compliance with national priorities, these mechanisms are not readily available to the MoH in Botswana. Currently there is limited financial flow from the MoH to the private healthcare providers. The private sector coordination mechanism is also weak and there are limitations in its capacity to monitor and evaluate private health sector services.

### Exerting Influence Through Regulation

While the MoH has done relatively well in facilitating development of health legislation, significant limitations were observed in the ability of the regulatory structures to enforce the legislation. While there is limited evidence on effective regulatory strategies, a command and control approach to regulation has generally been regarded as less effective.^54^ Increasingly, countries are exploring self*-*regulation which requires clear articulation of the role of non-governmental organizations (NGOs) such as professional organizations in the regulatory process, delegation of regulatory responsibilities, and building capacity of these organizations to undertake such responsibilities.^19,54^

### Coalition Building

Inadequate coordination, engagement and management of stakeholders are some of the challenges for the MoH in Botswana. While the MoH views the private health sector as a partner in the delivery of services, engagement mechanisms for this sector are still weak. Platforms that foster regular communication between the public and private sector are some of the success factors for effective private sector engagement.^55^ The organization of the private sector also affects its ability to engage with government. Where the private sector is more organized such as in Kenya and among NGOs in Uganda^55,56^ engagement with the public sector is more efficient.

The MoH’s lack of leadership and technical capacity contributed to weak stakeholder coordination and management. Where stakeholders are not well-coordinated, failure to create synergies among programs and interventions, high transaction costs and creation of uncoordinated service delivery structures are common features in the health system.^57^ Weak stakeholder management also creates opportunities for donor driven plans, a concern raised by some participants in this analysis. Effective leadership is essential for setting strategic directions through effective national health planning and building collective action towards achievement of national health goals.^58,59^ Employees with technical knowledge and experience bring a strong local context into partnerships ensuring that plans address national priorities.^58^

### Opportunities for Improved Stewardship

While there have been several challenges in the MoH’s ability to provide effective stewardship in the health system over the years, several developments in this area have the potential to improve performance (Table 4).

**Table 4:** Strategies for Improved Stewardship in Botswana Health Sector

**Stewardship Area**	**Strategies**	**Perceived Benefits**
Health policy development and implementation	• Takeover of PHC services from MLG - April 2010 • Establishment of the District Health System under MoH - ongoing • Revised National Health Policy approved - 2011 • Integrated Health Services Plan launched - 2012 • Development of health financing and resource allocation strategies - ongoing • Establishment of a national policy implementation office at the Office of the President	• More control; enhanced alignment of services with national policies • Improved efficiency in service delivery • Better guidance to the health system • Facilitation of policy implementation • Enhanced equity and sustainability • Improved accountability
Health regulation	• Establishment of the Health Inspectorate Department - 2012 • New Public Health Act passed - 2013	• Provision of oversight on the quality of health services • Improved regulation of health services
Coalition building	• Revival of the stakeholder forum - 2013 • Introduction of health sector reviews - 2013	• Improved public-private sector engagement • Enhanced joint planning, synergies in implementation and accountability

Abbreviations: MoH, Ministry of Health; MLG, Ministry of Local Government; PHC, primary healthcare.

These initiatives and strategies include revision of the National Health Policy and the Public Health Act, and development of other policy documents. The political will to improve policy implementation demonstrated by the Office of the President^60^ provides a good opportunity. With the positive developments highlighted in this section the MoH has the opportunity to turn around its performance in this area. However, it remains to be seen what impact these initiatives will have on the overall stewardship function of the MoH over time.

The use of WHO HSPAF to guide analysis of the various facets of health systems performance is a relatively new undertaking. The stewardship function, in particular, is grossly under-studied. This article, therefore, makes an important contribution in this area.

Generally there is limited consensus on methodologies for studying stewardship in health systems. Consequently, researchers adopt approaches that address specific contexts within which their studies are conducted. Similarly, this study focuses on the Botswana MoH. While important lessons can be drawn from this article, the findings cannot be generalized to other contexts. This study relied on the views of the MoH senior officers. However, the key challenge was the rapid turnover of these officers leading to overreliance on retired employees.

## Conclusion

The important contribution the WHO HSPAF makes in assessing health systems performance in general has been discussed elsewhere.^61^ Using this framework, the study of stewardship enabled identification of deficiencies in the resources, institutions, structures and processes necessary for undertaking the stewardship function. This information can be used by policy- and decision-makers to improve performance in this area.^62^

## Acknowledgements

The authors would like to thank the Botswana MoH management and staff for the support, and participants for their willingness. The research reported in this article was funded by a University of Otago Scholarship, for which the first author is grateful.

## Ethical issues

This study was approved by the University of Otago Ethics Committee, Dunedin, New Zealand and the Health Research and Development Committee of the Botswana MoH, Gaborone, Botswana.

## Competing interests

Authors declare that they have no competing interests.

## Authors’ contributions

OS, RG, PH, and PB were involved in the design of the study. OS undertook the study under the supervision of RG, PH, and PB. OS wrote the first draft of the article. RG, PH, and PB reviewed and commented on all drafts.

## Authors’ affiliations

^1^Ministry of Health, Gaborone, Botswana. ^2^Institute of Development Management, Gaborone, Botswana. ^3^Department of Preventive and Social Medicine, University of Otago, Dunedin, New Zealand. ^4^School of Health Sciences, University of Canterbury, Christchurch, New Zealand.

## Key messages

**Implications for policy makers**

- The Ministry of Health (MoH) in Botswana has challenges exercising its stewardship role. There is need to build the Ministry’s leadership capacity to oversee delivery of health services.
- Decentralization of health services within a weak policy and regulatory framework creates challenges in oversight. Appropriate policies and regulatory instruments guiding the role of different players in the health system are necessary. There is need to develop strategies to enhance the MoH’s regulatory capacity.
- Stakeholders play a critical role in health system development. The MoH in Botswana should optimize existing national private-public mechanisms to fully engage its stakeholders.

**Implications for public**

Stewardship is arguably the most important function of the health system because of its influence on other functions. This article demonstrates the need to strengthen the ability of the Ministry of Health (MoH) to direct, oversee and monitor the activities of the health system. More studies analyzing stewardship at various levels of health systems are necessary.

## References

[1] World Health Organization (WHO). The World Health Report 2000: Health systems: Improving performance. Geneva: WHO; 2000.

[2] World Health Organization (WHO). The Oaugadougou Declaration on Primary Health Care. Brazzaville: WHO Regional Office for Africa; 2008.

[3] World Health Organization (WHO). The Tallin Charter: Health systems for health and wealth. Copenhagen: WHO Regional Office for Europe; 2008.

[4] Kapoor N, Kumar D, Thakur N (2014). Core attributes of stewardship; foundation of sound health system. Int J Health Policy Manag.

[5] Siddiqi S, Masud TI, Nishtar S (2009). Framework for assessing governance of the health system in developing countries: gateway to good governance. Health Policy.

[6] Mikkelsen-Lopez I, Wyss K, De Savigny D (2011). An approach to addressing governance from a health system framework perspective. BMC International Health and Human Rights.

[7] Kaufmann D, Kraay A, Zoido-Lobaton P. Governance Matters. Washington: The World Bank; 1999.

[8] United Nations Development Programme (UNDP). Governance for sustainable human development: a UNDP policy document. New York: UNDP; 1997.

[9] Graham J, Amos B, Plumptre T. Principles for good governance in the 21st century. Policy brief no. 15. Ottawa: Institute on Governance; 2013.

[10] Islam M, ed. Health systems assessment approach: A how-to-manual. Submitted to the U.S. Agency for International Development in collaboration with Health Systems 20/20, Partners for Health Reformplus, Quality Assurance Project, and Rational Pharmaceutical Management Plus. Arlington, VA: Management Sciences for Health; 2007.

[11] Saltman RB, Ferroussier-Davis O (2000). The concept of stewardship in health policy. Bull World Health Organ.

[12] Management Science for Health. How to govern the health health sector and its institutions effectively. The eManager. 2013, No. 1.

[13] Seitio-Kgokgwe O. Organizational Structure of the Botswana Ministry of Health: Impact on Performance. Dunedin: Preventive and Social Medicine, University of Otago; 2012.

[14] World Health Organization (WHO). Everybody’s business: Strengthening health systems to improve health outcomes: WHO’s framework for action. Geneva: WHO; 2007.

[15] Travis P, Egger D, Davies P, Mechbal A. Towards better stewardship: concepts and critical issues. Geneva: WHO; 2002.

[16] Seitio-Kgokgwe O, Gauld R, Hill P, Barnett P (2014). Redesigning a Ministry of Health’s organizational structure: exploring implementation challenges through Botswana’s experiences. Int J Health Plan Manage.

[17] Seitio-Kgokgwe O, Gauld R, Hill P, Barnett P (2015). Development of the national health information systems in Botswana: pitfalls, prospects and lessons. Online Journal of Public Health Informatics.

[18] Roberts MJ, Hsiao W, Berman P, Reich MR. Getting Health Reform Right: A Guide to Improving Performance and Equity. New York: Oxford University Press; 2004.

[19] Bennett S, Hanson K, Kadama P, Montagu D. Working with the non-state sector to achieve public health goalls. Geneva: WHO; 2005.

[20] Nishtar S (2004). Public-private ‘partnerships’ in health- a global call for action. Health Res Policy Syst.

[21] Central Statistics Office (CSO). Population and housing census: preliminary results. Gaborone: CSO; 2011.

[22] Ministry of Health (MoH). National health accounts. Gaborone: MoH; 2012.

[23] Central Statistics Office (CSO). Health statistics 2000. Gaborone: CSO; 2003.

[24] Ministry of Finance and Development Planning (MFDP). National Development Plan 9: 2003/4-2008/9. Gaborone: MFDP; 2003.

[25] Ministry of Finance and Development Planning (MFDP). National Development Plan 1970/75. Gaborone: MFDP; 1970.

[26] Ministry of Health (MoH). A situational analysis of health insurance (Medical Aid) in Botswana’s formal sector: A health financing study. Gaborone: MoH; 2004.

[27] Ministry of Health (MoH). Republic of Botswana National Health Accounts. Gaborone: MoH; 2006.

[28] Yin RK. Case Study Research: Design and Methods. 3rd ed. London: Sage Publications; 2003.

[29] Creswell JW. Research Design: Qualitative, Quantitative and Mixed Methods Approaches. 2nd ed. London: Sage Publications Inc; 2003.

[30] Teddlie C, Tashakkori A. Foundations of Mixed Methods Research: Integrating Quantitative and Qualitative Approaches in the Social and Behavioural Sciences. Thousand Oaks: Sage; 2009.

[31] Babbie E, Mouton J. The Practice of Social Research. Cape Town: Oxford University Press Southren Africa; 2001.

[32] Miles MB, Huberman AM. Qualitative Data Analysis. 2nd ed. Thousands Oaks: Sage Publications; 1994.

[33] Ministry of Health (MoH). Corporate performance plan 2000-2005. Gaborone: MoH; 2000.

[34] Ministry of Health (MoH). A review of the organizational structure of the MoH in Botswana: Final report Gaborone: MoH; 2002.

[35] British Council. A review of the organisational structure of the MoH in Botswana: Discussion document stakeholders meeting 19 June 2002. Gaborone; MoH: 2002.

[36] Ministry of Health (MoH). National Health Policy. Gaborone: MoH; 1995.

[37] Molutsi P, Lauglo M. Decentralisation and health systems performance: the case of Botswana. Gaborone: MoH; 1994.

[38] GoB. Public Health: Chapter 63:01. Gaborone 1981.

[39] GoB. Private Hospitals and Nursing Homes Act. Gaborone 1989.

[40] GoB. Drugs and Related Substances Act No. 18 of 1992. Gaborone 1992.

[41] GoB. Botswana Health Professions: Chapter 61:02. Gaborone 2001.

[42] GoB. Nurses and Midwifes: Chapter 61:03. Gaborone 1997.

[43] Ministry of Finance and Development Planning (MFDP). National Development Plan 8: 1997/98-2002/03. Gaborone: MFDP; 1997.

[44] Ministry of Health (MoH). National health service situational analysis report. Gaborone: MoH; 2009.

[45] Bossert T, Beauvais J (2002). Bossert T, Beauvais JDecentralization of health systems in Ghana, Zambia, Uganda and the Philippines: a comparative analysis of decision space. Health Policy Plan.

[46] Danishevski K, Balabanova D, McKee M, Atkinson S (2006). The fragmentary federation: experiences with the decentralized health system in Russia. Health Policy Plan.

[47] Bossert T, Chitah MB, Browser D (2003). Decentralization in Zambia: resource allocation and district performance. Health Policy Plan.

[48] Tenbensel T. Health targets. Health Policy Monitor. 2007. Survey no: 10.

[49] Mills A. Decentralization concepts and issues: a review. In: Mills A, Vaughan JP, Smith DL, Tabibzadeh I, eds. Health System Decentralization: Concepts, Issues, and Country Experiences. Geneva: WHO; 1990.

[50] Buse K, Mays N, Walt G. Making Health Policy. London: Open University Press; 2005.

[51] Hanson K, Berman P (1998). Private health care provision in developing countries: a preliminary analysis of levels and compositions. Health Policy Plan.

[52] McPake B, Anne M (2000). What can we learn from international comparisons of health systems and health system reform?. Bull World Health Organ.

[53] Harding A, Preker A. Understanding organizational reforms The corporatization of public hospitals. Washington DC: The World Bank; 2000.

[54] Ensor T, Weinzierl S (2007). Regulating health care in low- and middle-income countries: Broadening the policy response in resource constrained environments. Soc Sci Mede.

[55] Balabanova D, Oliveira-Cruz V, Hanson K. Health sector governance and implications for the private sector. Washington DC: Result for Development Institute; 2008.

[56] Barnes J, O’Hanlon B, Feeley F, Mckeon K, Gitonga N, Decker C. Kenya private health sector assessment. Bethesda, MD: Private sector partnership-One Project, Abt Associates, Inc; 2009.

[57] Caines K. Best Practice Principles for Global Health Partnership Activities at Country Level. http://www.eldis.org/go/home&id=20999&type=Document#.V0m2m-RdTfY. Published 2005.

[58] Cahill K, Fleming D, Conway M, Gupta S. Global Health Partnerships: Assessing Country Consequences. WHO/The World Bank; 2005.

[59] Downs TJ, Larson HJ (2007). Achieving Millennium Development Goals for health: Building understanding, trust and capacity to respond. Health Policy.

[60] Seretse Khama IK. Speech by H.E. Lieutenant General Seretse Khama Ian Khama-President of the Republic of Botswana and BDP Party President at the National Council 22/5/10. Tautona Times. May 2010.

[61] Seitio-Kgokgwe O, Gauld R, Hill P, Barnett P (2014). Assessing performance of Botswana’s public hospital system: the use of the World Health Organization Health System Performance Assessment Framework. Int J Health Policy Manag.

[62] Rubin HR, Pronovost P, Diette GB (2001). The advantages and disadvantages of process-based measures of health care quality. Int J Qual Health Care.

